# Effect of CTP-mediated PTEN on 5637 bladder cancer cells and the underlying molecular mechanism

**DOI:** 10.1186/s12894-022-01152-y

**Published:** 2022-12-10

**Authors:** Bei Yu, Yuan Huang, Yue Yang, Haifeng Hu, Jin Yang

**Affiliations:** 1 Urological Department, The Affiliated Hospital of Chengdu University, Chengdu, Sichuan China; 2 Department of Clinical Laboratory, The Affiliated Hospital of Chengdu University, Chengdu, Sichuan China

**Keywords:** PTEN, CTP-PTEN, Bladder cancer, Carcinostasis

## Abstract

**Objective:**

The aim of the present study was to explore the effect of cytoplasmic transduction peptide (CTP)-phosphatase and tensin homolog (PTEN) on the proliferation, cell cycle, apoptosis, migration and invasion of bladder cancer cells and the underlying molecular mechanism.

**Methods:**

A eukaryotic expression vector, pTT5-CTP-PTEN, was constructed. The constructed vector was transfected into HEK 293-6E cells to express a fusion protein, CTP-PTEN. The fusion protein was purified. 5637 bladder cancer cells were cocultured with purified CTP-PTEN fusion protein. Target gene expression, protein expression, cell proliferation, cell cycle, apoptosis, cell invasion and cell migration were examined by reverse transcription polymerase chain reaction (RT-PCR), western blot, MTT assay, flow cytometry, Transwell assay, and cell scratch assay, respectively.

**Results:**

Both PTEN and CTP-PTEN fusion protein inhibited the proliferation, cell cycle, invasion and migration of bladder cancer cells and promoted the apoptosis of bladder cancer cells. The effect of CTP-PTEN was more significant.

**Conclusions:**

The fused expression of CTP and PTEN significantly increased the penetrability of the tumor suppressor gene PTEN into cancer cells. The CTP-PTEN fusion protein exhibited a significant carcinostatic effect on 5637 bladder cancer cells.

**Supplementary Information:**

The online version contains supplementary material available at 10.1186/s12894-022-01152-y.

## Introduction

Bladder cancer is a common malignancy of the urinary system. There are approximately 440,000 new cases of bladder cancer worldwide each year, and approximately 160,000 patients die from bladder cancer each year [1]. Males have an approximately 1.1% chance of developing bladder cancer in their lifetime. For women, the risk is approximately 0.27% [2, 3]. The most common manifestations of bladder cancer are gross hematuria and microscopic hematuria. Surgery is an effective treatment for early bladder cancer [4]. However, there is a lack of specific treatments for most advanced and moderately advanced bladder cancers.

The gene phosphatase and tensin homolog (PTEN) is one of the most widely studied tumor suppressor genes [5, 6]. It was first discovered in 1997 during the study of the chromosome 10q23 locus [7]. In a hypomorphic allele mouse model, the downregulation of PTEN expression significantly increased susceptibility to cancer, and the regulation of the function and stability of PETN affected the occurrence and development of cancer [8]. Due to low transfection efficiency, the effect of gene therapy has not been fully realized. The key to improving the effect of gene therapy lies in the carrier system.

Cytoplasmic transduction peptide (CTP) is an endocytic pathway-independent cytoplasmic transduction peptide [9]. It mediates the penetration of proteins through the cell membrane and the localization of proteins specifically in the cytoplasm. As a novel carrier system for tumor gene therapy, CTP shows unique advantages in tumor research [10]. Fusion expression of the tumor suppressor gene PTEN and CTP allows CTP to mediate the penetration of the purified exogenous PTEN protein through the cell membrane and the localization of PTEN in the cytoplasm. This strategy may successfully achieve the “endogenization” of exogenous proteins, which not only avoids the series issues caused by traditional gene transfection (such as gene mutation and loss) but also inhibits or kills tumor cells. This study explored the effects of CTP-PTEN on the migration and invasion of the 5637 bladder cancer cell line to provide a theoretical basis for new treatment methods for bladder cancer.

## Materials and methods

### Materials

Escherichia coli DH5α and HEK 293-6E cells were previously cryopreserved in our laboratory. The pTT5 plasmid was purchased from Nanjing Genscript Biotechnology Co., Ltd. The CTP-PTEN fusion gene was synthesized by Nanjing Genscript Biotechnology Co., Ltd. PTEN protein was purchased from Abcam (ab157087). Plasmid extraction kits, PCR kits, restriction endonucleases EcoR I and Hind III, DNA ligation kits, and DNA markers were purchased from TaKaRa Bio., Inc. The reagents for determining protein concentrations were purchased from Bio-Rad Laboratories. Endotoxin-free plasmid extraction kits and gel recovery kits were purchased from Omega. RIPA lysis buffer and hypersensitive ECL chemiluminescence kits were purchased from Shanghai Beyotime Biotechnology Co., Ltd. FreeStyle 293 cell culture medium and Lipofectamine2000 were purchased from Invitrogen Corporation. Rabbit anti-PTEN monoclonal antibody was purchased from Abcam (ab32199). Goat anti-rabbit IgG-HRP was purchased from Santa Cruz Biotechnology, Inc. 5637 bladder cancer cells were obtained from the Institute of Basic Medicine, Chinese Academy of Medical Sciences. Thiazole blue (methyl thiazolyl tetrazolium, MTT) was purchased from Nanjing Vazyme Biotechnology Co., Ltd. Annexin V-fluorescein isothiocyanate (FITC) and propidium iodide (PI) kits were purchased from Nanjing Senbeijia Biotechnology Co., Ltd. RNA extraction kits, reverse transcription kits, and qRT-PCR kits were all purchased from Chengdu Foregene Biotechnology Co., Ltd. BD Transwell chambers were purchased from Beijing Mingyang Kehua Biotechnology Co., Ltd., China.

### Methods

#### Synthesis of the CTP-PTEN fusion gene

The CTP-PTEN fusion gene was synthesized based on the GenBank accession number NM_000314 and the CTP gene sequence (TACGGAAGAAGGGCTAGACGAAGAAGACGAAGA) [11]. EcoR I and Hind III restriction sites were introduced at each end of the fusion gene.

#### Construction and verification of the pTT5-CTP-PTEN recombinant plasmid

The pTT5 plasmid was double digested with EcoR I and Hind III. The volume of the enzymatic digestion system was 50 μl: target gene or vector, 30 μl; EcoR I, 1.2 μl; Hind III, 1.2 μl; 10 × M Buffer, 2 μl; and sterilized deionized water, 15.6 μl. The digestion system was kept in a 37 °C water bath for 2 h. Subsequently, 3 μl of the enzyme digestion product was subjected to agarose gel electrophoresis (1.2%). The remaining product was purified using a PCR product purification kit. To ligate the digested pTT5 vector with the CTP-PTEN fusion gene, a 20-μl ligation system was prepared based on a 1:5 molar ratio of the digested vector-to-the target gene and T4 DNA ligase: target gene, 7.7 μl; vector, 8.3 μl; 10 × T4 Buffer, 2 μl; and T4 DNA ligase, 2 μl. The ligation system was kept at 4 °C overnight and then incubated in a 70 °C water bath for 10 min to inactivate the T4 DNA ligase. The product was stored for future assays. pTT5-CTP-PTEN was added to 100 μl of freshly prepared competent cells. After incubation on ice for 30 min, the test tube containing the cells was placed in a 42 °C water bath and heat-shocked for 90 s. The test tube was then taken out and quickly placed in an ice bath for 2 min. Subsequently, 400 μl of LB liquid medium was added, and the cells were resuscitated slowly at 37 °C for 1 h. The cells were then spread on an LB plate containing 100 μg/ml ampicillin (Amp) and incubated at 37 °C overnight. The resulting colonies were picked and cultured at 37 °C overnight in 10 ml of LB containing 100 μg/ml Amp. The plasmid was extracted using a plasmid extraction kit. The positive clones were screened by double enzyme digestion and verified by sequencing.

#### The expression, identification and purification of CTP-PTEN fusion protein in HEK 293-6E cells

Cryopreserved HEK 293-6E cells were resuscitated and cultured in FreeStyle 293 medium. The cells were transferred into 6-well plates and divided into 3 groups: group A was transfected with liposomes, group B was transfected with liposome-encapsulated empty plasmid, and group C was transfected with the liposome-encapsulated recombinant plasmid pTT5-CTP-PTEN. Four micrograms of purified pTT5-CTP-PTEN recombinant plasmid or empty pTT5 vector were used. The vectors were transfected into HEK 293-6E cells using Lipofectamine 2000 in accordance with the manufacturer’s instructions. After transfection, the different groups of HEK 293-6E cells were cultured for 48 h. At 48 h after transfection, the 6-well plates containing HEK 293-6E cells were placed on ice. The culture medium was discarded, and the HEK 293-6E cells were washed 3 times with prechilled PBS. The cells were then scraped off with a cell scraper. Suspensions of the liposome-transfected cells, the empty plasmid-transfected cells and the recombinant plasmid-transfected cells were transferred to centrifuge tubes, mixed thoroughly with 50 μl of protein lysis buffer containing protease inhibitors, and placed on ice for 30 min. After centrifugation at 13,000 r/min and 4 °C for 20 min, 5 μl of supernatant was collected for protein quantification. Subsequently, 40 µg of protein was mixed with 5 × SDS-PAGE loading buffer, denatured at 100 °C for 5 min, separated by 10% SDS-PAGE, and transferred to membranes at 4 °C and 70 V for 3.5 h. The membranes were washed once with TBST, blocked at room temperature for 2 h in 5% nonfat milk, and incubated with anti-PTEN antibody at 4 °C overnight. The membranes were then washed 3 times with TBST, incubated with goat anti-rabbit secondary antibody at room temperature for 2 h, and again washed 3 times with TBST. Chromogenic solutions A and B in the ECL western blot (WB) detection kit were mixed in equal volumes, and the membranes were allowed to fully contact the mixture. The membranes were then placed in an ECL luminometer and exposed for 1 min. Protein expression was examined using the ECL imaging system, and the expression of the internal reference β-actin was also examined. After successful expression in cells, the protein was purified by Ni sepharose fast flow affinity chromatography using 5 solutions, i.e., A, B, C, D, and E. The solutions were as follows: A: 20 mmol/L sodium phosphate buffer (PB), 500 mmol/L sodium chloride, and 5 mmol/L imidazole, pH 7.4; B: 20 mmol/L PB, 500 mmol/L sodium chloride, and 20 mmol/L imidazole, pH 7.4; C: 20 mmol/L PB, 500 mmol/L sodium chloride, and 60 mmol/L imidazole, pH 7.4; D: 20 mmol/L PB, 500 mmol/L sodium chloride, and 100 mmol/L imidazole, pH 7.4; and E: 20 mmol/L PB, 500 mmol/L sodium chloride, and 300 mmol/L imidazole, pH 7.4. First, the collected culture medium was centrifuged (4750 × g) at 4 °C for 8 min, and the precipitate was removed. The supernatants were concentrated using a 6 × 10^3^ hollow fiber column and filtered with a plate filter. After preequilibration of the affinity chromatography column with solution A, the filtered supernatants were slowly loaded at a rate of 3 mL/min. The unbound contaminant proteins were washed away using solutions B, C, and D, while the target protein was eluted using solution E. The eluted target protein was dialyzed against a biological semipermeable membrane for 48 h to fully remove the salt and imidazole (the dialysate was PBS, pH 7.4). The purified target protein was subjected to Coomassie brilliant blue staining and WB analysis.

#### Cell culture, transfection and grouping

The 5637 bladder cancer cells were provided by Cell Bank of Chinese Academy of Sciences. 5637 bladder cancer cells were subjected to routine culture in Dulbecco's Modified Eagle's Medium (DMEM) containing 10% fetal bovine serum. The 5637 cells were randomly divided into the 5637 group (untreated cells), the PTEN group (5637 bladder cancer cells cocultured with PTEN), and the CTP + PTEN group (5637 bladder cancer cells cocultured with CTP-PTEN).

#### Examination of the cellular expression of Akt, mechanistic target of rapamycin (mTOR) and phosphoinositide 3-kinases (Pi3k) by quantitative polymerase chain reaction (qPCR)

The untreated 5637 cells and cells treated by PTEN and CTP-PTEN were collected by centrifugation. Total RNA was extracted by the TRIzol method, and cDNA was synthesized from the extracted RNA using a reverse transcription kit. The relative expression levels of Akt, Mtor and Pi3k were examined by qPCR. Glyceraldehyde 3-phosphate dehydrogenase (GAPDH) was used as the internal control. The expression of Akt, Mtor and Pi3k was calculated by the 2^−ΔΔCt^ method. The qPCR reaction conditions were as follows: 95 °C for 5 min; 95 °C for 30 s, 60 °C for 30 s, and 72 °C for 1 min, for 40 cycles; and final extension at 72 °C for 5 min.

#### WB analysis of the protein expression of Akt, p-Akt, mTOR, p-mTOR and Pi3k

After 48 h of coculture, the cells were collected by centrifugation. Total cellular protein were extracted using a kit, and the protein concentration was quantified by the bicinchoninic acid (BCA) method. Total protein was mixed with 5 volumes of loading buffer, boiled for 10 min, and then centrifuged at 12,000 rpm for 5 min. The resulting supernatants were collected, and the samples were loaded. After electrophoresis, the protein was transferred to membranes. The transfer was carried out at a 4 °C. The membranes were then blocked with 5% skimmed milk and incubated with diluted primary antibody at 4 °C overnight. The primary p-Akt (ab81283) and Pi3k (ab40776) antibodies were purchased from Abcam (Cambridge, MA; USA); Akt (#9272), mTOR (#2983) and p-mTOR (#5536) from Cell signaling Technology; GAPDH (17552-1-AP) was from Proteintech Group, Inc. The next day, the membranes were incubated with diluted secondary antibody at room temperature for 50 min. Image development, fixation and exposure were performed in a dark room. The gray values of the protein bands were determined using ImageJ.

#### Examination of PIP3 content by the ELISA assay

The PIP3 content in 5637 cells line was determined according to the instructions of the Human Phosphatidylinositol Triphosphate (PIP3) ELISA Kit (LunChangShuoBiotech, Co., Ltd.).

#### Examination of cell proliferation by the MTT assay

Cells were collected after 48 h of coculture. The cells were then seeded into 96-well plates at a density of 5 × 10^5^ cells/mL. After the addition of 20 μL of MTT solution and 150 μL of dimethylsulfoxide (DMSO), the absorbance of cells was measured at the wavelength of 490 nm. Cell survival rate = A490 of sample/A490 of control × 100%.

#### Examination of cell cycle and apoptosis by flow cytometry

After 48 h of cocultivation, cells were collected and subjected to flow cytometric analysis using an Annexin V-FITC/PI kit. The flow cytometry results were analyzed.

#### Examination of cell migration with the cell scratch assay

After 48 h of cocultivation, cells were scratched with a 200-μL pipette tip. The cells were then rinsed with PBS and then cultured in serum-free medium. The scratch assay was conducted at 0 h and 48 h, and images were taken. The scratch distance was measured using ImageJ software, and the cell migration rate was calculated. The assay was repeated 3 times. The statistical graphs of the scratch migration rates were plotted for each group of cells using GraphPad Prism 7.0.

#### Examination of cell invasion with the Transwell assay

Cell invasion was examined using small chambers coated with Matrigel. The cells to be tested were adjusted to a density of 5 × 10^5^ cells/mL after 48 h of coculture and cultured for another 24 h in serum-free medium. Subsequently, 200 μL of cells was aspirated and spread on the surface of the polycarbonate membrane in the upper chamber (the membrane was wet). Subsequently, 600 μL of serum-containing medium was added to the lower chamber. The chambers were placed in a 37 °C incubator and incubated for 24 h. Crystal violet staining solution (1 g/L) and methanol fixative were prepared. The residual cells on the upper surface of the upper chamber membrane were removed. The membranes were fixed in methanol for 30 min and then stained for 15 min in the dye solution. After staining, the membranes were mounted and examined under a microscope. Three microscopic fields of view were selected and imaged. The number of cells in the fields were counted, and the average value was determined. Three replica chambers were set up for each sample, and the experiment was repeated 3 times.

#### Statistical analysis

All experimental data were analyzed using GraphPad Prism 7.0. The measurement data are presented as x ± s. Comparisons between multiple groups of data were performed using one-way analysis of variance (ANOVA), while comparisons between 2 groups of data were conducted using the *t* test. A P value less than 0.05 indicated that the difference was statistically significant.

## Results

### The successful construction of the recombinant plasmid pTT5-CTP-PTEN was verified by double enzyme digestion and sequencing

**Fig. 1:**
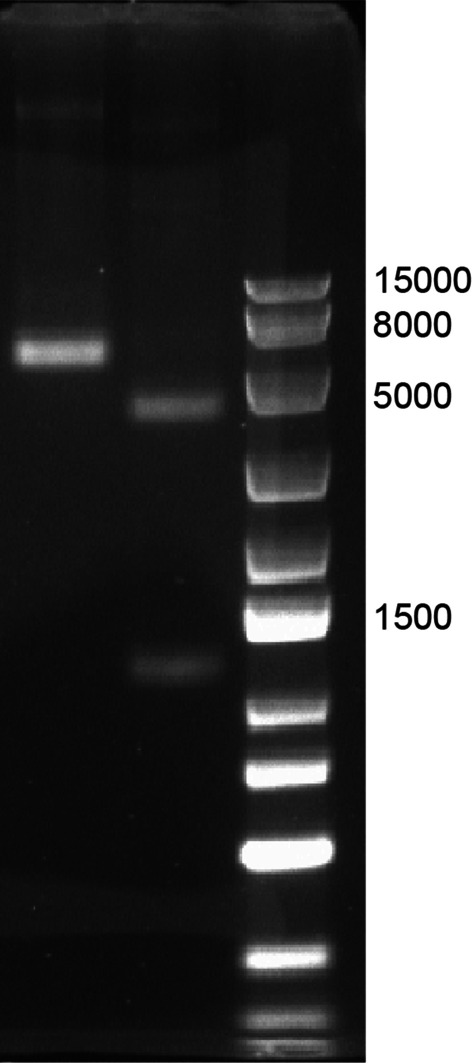
The recombinant plasmid pTT5-CTP-PTEN was identified by EcoRI and HindIII; Lane 1: pTT5-CTP-PTEN; Lane 2: pTT5-CTP-PTEN Digested with EcoRI-HindIII; Lane M: DNA Marker

Double enzyme digestion of the coding gene of pTT5-CTP-PTEN with EcoR I and Hind III generated 2 distinct bands: a vector fragment (approximately 4401 bp) and a target gene fragment (approximately 1293 bp) (see Fig. 1). The constructed recombinant plasmid was sequenced. It was confirmed that the sequence of the recombinant plasmid was exactly the same as the designed sequence.

### WB confirmed that the CTP-PTEN fusion protein was successfully expressed in and purified from HEK 293-6E cells

**Fig. 2:**
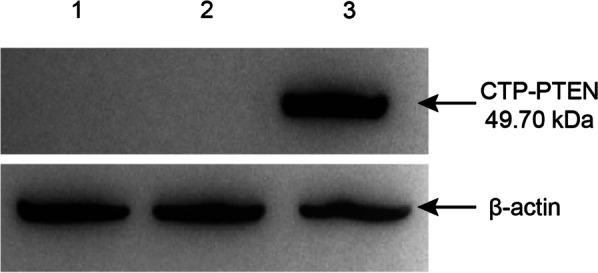
Western blot analysis of CTP-PTEN expression in HEK 293-6E cell. Lane 1: HEK 293-6E cell lysate supernatant from blank control; Lane 2: HEK 293-6E cell lysate supernatant from post-transfection of pTT5; Lane 3: HEK 293-6E cell lysate supernatant from post-transfection of pTT5-CTP-PTEN

Various groups of cells were collected after 48 h of cocultivation, and the expression of the CTP-PTEN fusion protein in these cells was examined by WB. WB detected a clear positive band in the group transfected with the pTT5-CTP-PTEN recombinant plasmid, demonstrating that the CTP-PTEN fusion protein was successfully expressed in HEK 293-6E cells. In contrast, no positive band was detected in the liposome-transfected group or in the group transfected with liposome-encapsulated empty plasmid (Fig. 2).

### CPT-PTEN and PTEN inhibited cell proliferation

**Fig. 3:**
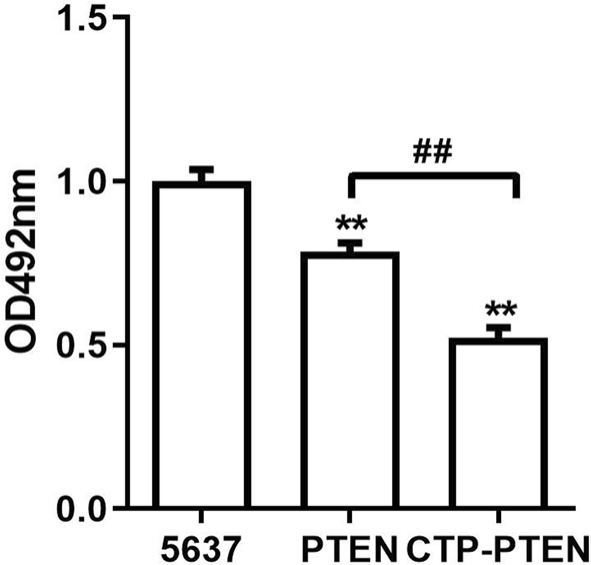
The effect of CPT-PTEN and PTEN on proliferation of 5637 cell lines

As shown in Fig. 3, the proliferation of 5637 cells was significantly inhibited by CPT-PTEN and PTEN (*P* < 0.01). Moreover, CTP-PTEN inhibited the proliferation of 5637 cells more significantly than did PTEN (*P* < 0.01).

### CPT-PTEN and PTEN promoted the apoptosis of 5637 cells

**Fig. 4:**
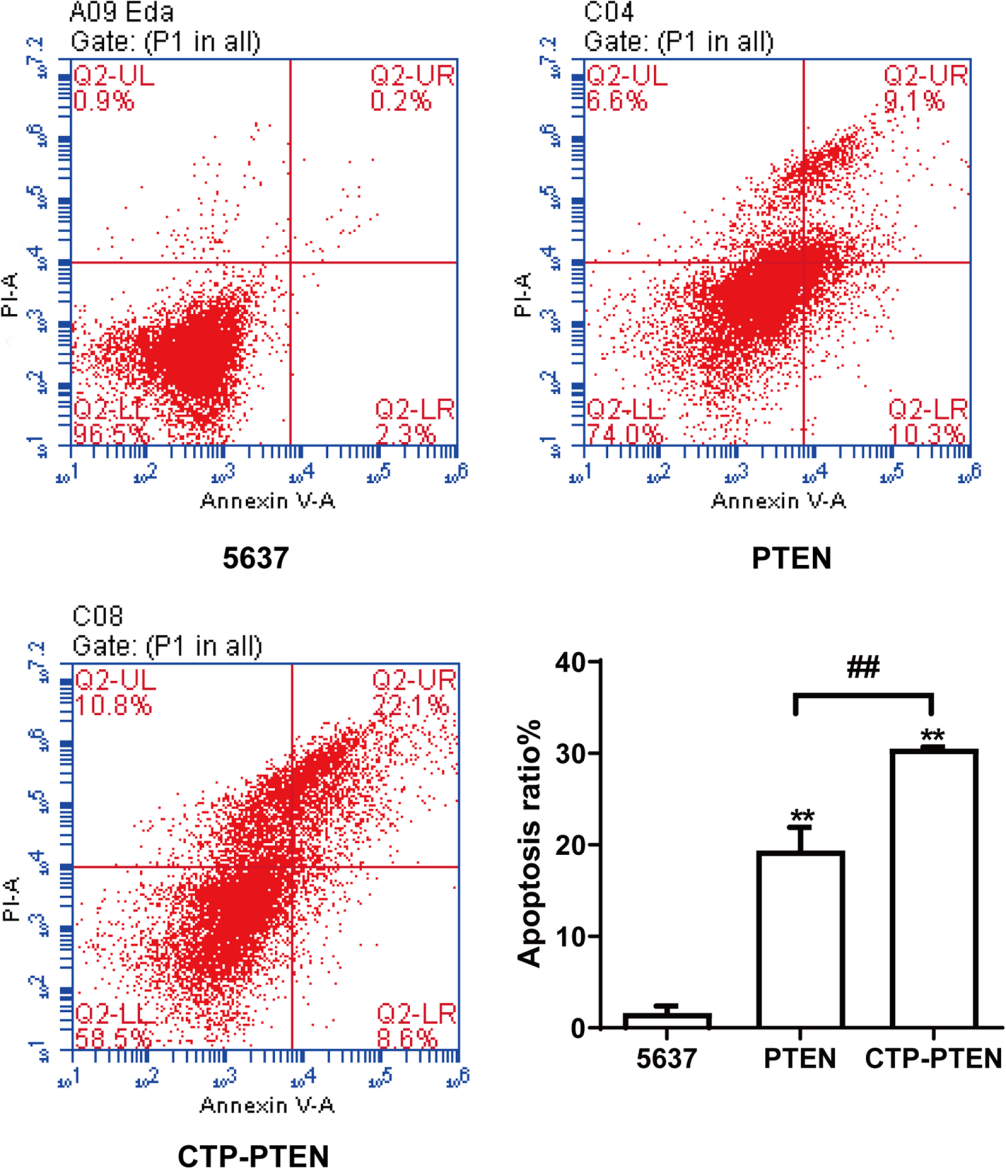
CTP-PTEN and PTEN promoted apoptosis of 5637 cells

The apoptosis of cancer cells was analyzed by flow cytometry (Fig. 4). Compared with the 5637 group, apoptosis was significantly increased in the PTEN group and the CTP-PTEN group (*P* < 0.01). Moreover, the apoptotic rate was significantly higher in the CTP-PTEN group than in the PTEN group (*P* < 0.01).

### The effect of CTP-PTEN and PTEN on the cell cycle

**Fig. 5:**
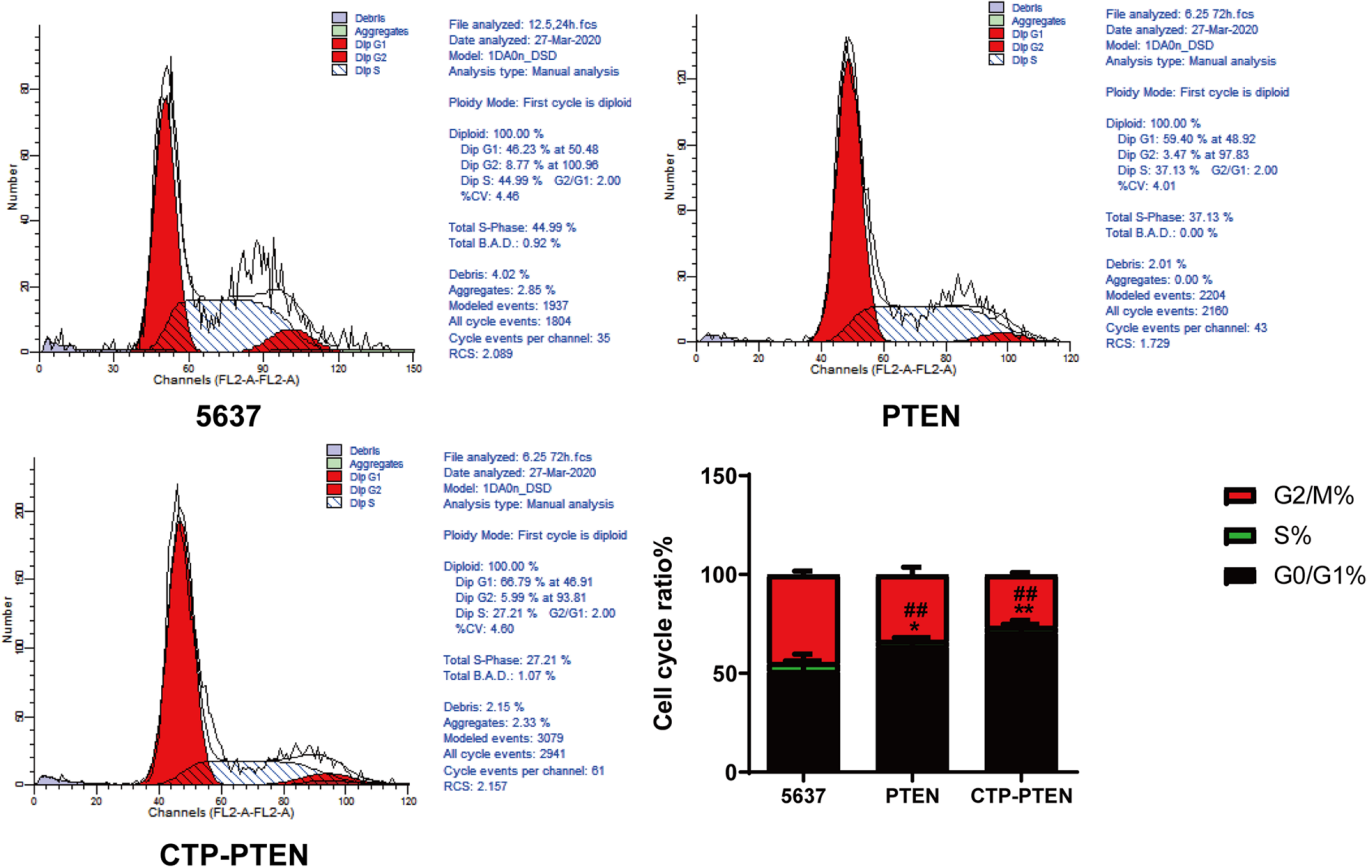
The effect of CTP-PTEN and PTEN on cell cycle of cell 5637

Compared with that in the 5637 group, the percentage of 5637 cells in S phase was significantly reduced in the CTP-PTEN group (*P* < 0.01) and the PTEN group (*P* < 0.05). Moreover, the percentage of S-phase 5637 cells was significantly lower in the CTP-PTEN group than in the PTEN group (*P* < 0.01) (Fig. 5). Therefore, CTP-PTEN and PTEN significantly inhibited cell cycle progression from G1 phase to G2 phase.

### The effect of CTP-PTEN and PTEN on the cell migration rate

**Fig. 6:**
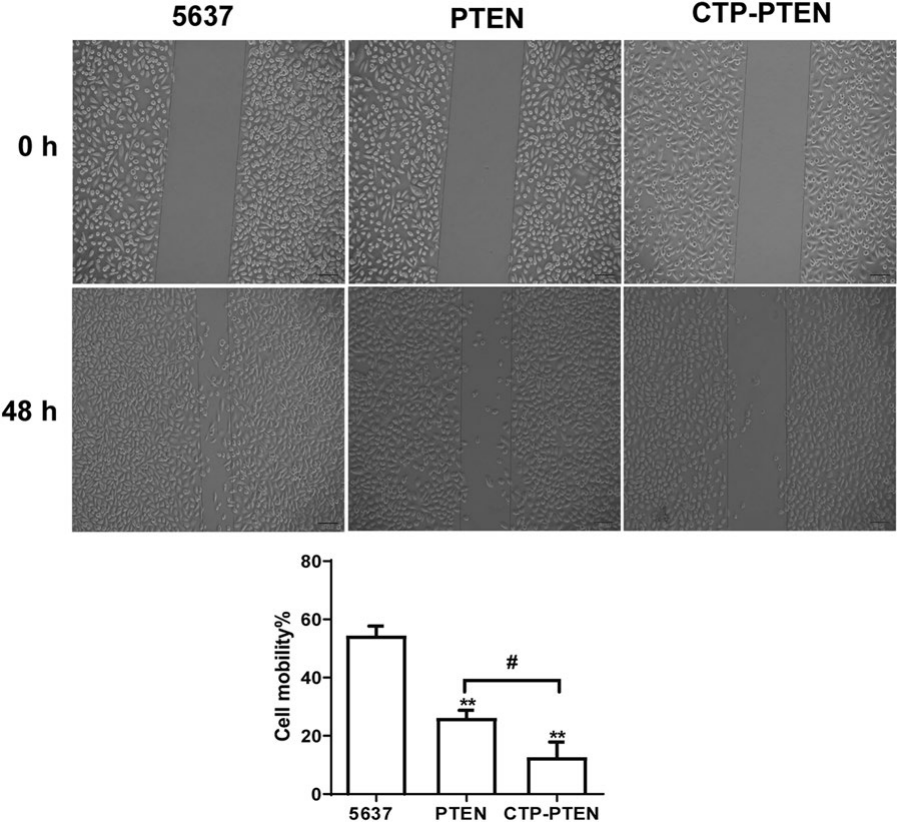
The effect of CTP-PTEN and PTEN on migration of cell line 5637

At 48 h after transfection, compared with that in the control group, cell migration was significantly inhibited in the CTP-PTEN and PTEN groups (*P* < 0.01). Compared with that in the PTEN group, cell migration was significantly inhibited in the CTP-PTEN group (*P* < 0.05) (Fig. 6).

### CTP-PTEN and PTEN inhibited cell invasion

As shown in Additional file 1: Figure S-1, compared with that in the 5637 group, cell invasion was significantly inhibited in the CTP-PTEN and PTEN groups (*P* < 0.01). Compared with that in the PTEN group, cell invasion was significantly inhibited in the CTP-PTEN group (*P* < 0.01).

### CTP-PTEN and PTEN inhibited the expression of the target genes in 5637 cells

As shown in Additional file 1: Figure S-2, compared with that in untreated 5637 cells, the expression of Akt, Mtor and Pi3k was significantly inhibited in the CTP-PTEN and PTEN groups (*P* < 0.01, *P* < 0.05). Compared with that in the PTEN group, the expression of Mtor and Pi3k was significantly inhibited in the CTP-PTEN group (*P* < 0.01, *P* < 0.05).

### The effect of CTP-PTEN and PTEN on the expression and phosphorylation of the target proteins

As shown in Additional file 1: Figure S-3, compared with that in 5637 cells, the expression of Akt protein was significantly inhibited in the CTP-PTEN treatment group (*P* < 0.01). In addition, the expression of mTOR and PI3K proteins and the phosphorylation of Akt and mTOR were significantly inhibited in the cells treated with CTP-PTEN and PTEN (*P* < 0.01, *P* < 0.05). Compared with that in the PTEN group, the expression of Akt, mTOR and PI3K and the phosphorylation of Akt and mTOR were significantly inhibited in the CTP-PTEN group (*P* < 0.01).

### The effect of CTP-PTEN and PTEN on the content of PIP3 in 5637 cells

As shown in Additional file 1: Figure S-4, compared with that in 5637 cells, the content of PIP3 was significantly reduced in the CTP-PTEN treatment group (*P* < 0.01). In addition, the content of PIP3 were significantly reduced in the cells treated with PTEN (*P* < 0.01). Compared with that in the PTEN group, the content of PIP3 were significantly decreased in the CTP-PTEN group (*P* < 0.01).

## Discussion

As one of the most widely studied tumor suppressor genes, the PTEN gene has broad research prospects. However, due to low transfection efficiency, the effect of gene therapy has not been fully achieved. The key to improving the effect of gene therapy lies in the carrier system [12]. Therefore, the development of an efficient, specific and safe carrier system that is able to directly transfer biologically effective proteins into tumor cells (especially living tissue cells) to inhibit or kill tumors has become a research hotspot in China and other countries.

The PTEN gene plays an important carcinostatic effect in a variety of tumor cells. Studies have shown that the PTEN gene is inactivated in a variety of malignant tumors (such as bladder cancer, prostate cancer, endometrial cancer and gastric cancer) due to deletion, mutation, or reduced transcription [13–16]. PTEN has phosphatase activity. PTEN downregulates the phosphorylation levels of the tyrosines in focal adhesion kinase (FAK) and p130 through dephosphorylating FAK. By inhibiting FAK activity, PTEN reduces integrin-mediated cell spreading and local adhesion formation, thereby blocking cell migration and inhibiting tumor invasion and metastasis [17]. In addition, PTEN inhibits the activation of extracellular signal-regulated kinase (ERK) and RAS and the phosphorylation of Shc in the upstream mitogen-activated protein kinase (MAPK) pathway, thereby regulating the MAPK/ERK signaling pathway. PTEN also inhibits the phosphorylation of MAPK, arrests the cell cycle in G1 phase, and inhibits the growth of tumor cells [18]. PTEN has not only protein phosphatase activity but also lipid phosphatase activity. The carcinostatic effect of PTEN is mainly achieved through its lipid phosphatase activity, which dephosphorylates the intracellular second messengers phosphatidylinositol 3,4-diphosphate (PIP2) and phosphatidylinositol 3,4,5-triphosphate (PIP3) and reduces the level of PIP3. PIP3 is the catalytic product of PI3K. A reduction in PIP3 level negatively regulates the PI3K/protein kinase B (PKB)/Akt signal transduction pathway, induces the activity of caspase-9 and P27, and inhibits the activity of cyclin-dependent kinase (CDK). As a result, PTEN arrests the cell cycle in G1 phase and mediates apoptosis, thereby exerting a carcinostatic effect [19]. PTEN also participates in cell proliferation, differentiation, apoptosis and metabolism by regulating the PI3K/Akt/mTOR signaling pathway [20]. PTEN negatively regulates the downstream effector molecules of this pathway, thereby inhibiting the synthesis of ribosomal proteins and translational regulatory proteins. As a result, PTEN is able to regulate protein synthesis, control cell growth and size, and support cell growth and survival.

CTP is a recently reported transduction peptide that is able to efficiently carry proteins through the cell membrane. CTP exerts its transduction function via an endocytosis-independent mechanism. In addition, CTP exhibits a significant cytoplasmic localization preference. It is expected that the utilization of CTP will facilitate the “endogenization” of exogenous proteins. Therefore, CTP has a high potential to become an efficient carrier for gene therapy [9]. Kim et al. recently used CTP as a carrier and successfully constructed a CTP-PD1 fusion protein for the treatment of glioblastoma [21]. More importantly, in terms of biosafety, traditional gene therapy has potential hazards, such as damage to genetic material and tumorigenicity. In contrast, after the removal of the nuclear localization signal, CTP cannot localize to the nucleus, preventing the possibility of damaging genetic material in the nucleus. The method that involves the fusion of proteins with CTP, eukaryotic expression of the fusion protein and subsequent transduction of the purified recombinant protein into target cells is effective and safe. At present, several studies have demonstrated that CTP efficiently carries target proteins and localizes the proteins in the cytoplasm [22–24].

Therefore, we fused the tumor suppressor gene PTEN with CTP. The purified exogenous PTEN protein directly penetrated the cell membrane and localized in the cytoplasm under the mediation of CTP. As the result, “endogenization” of the exogenous protein was successfully achieved. Such a strategy not only avoided a series of problems (such as gene mutation and loss) caused by the traditional gene transfection method but also inhibited or killed tumor cells. By directly and effectively transferring biologically effective proteins into tumor cells, the abovementioned difficulties can be overcome, and the therapeutic effect can be greatly improved.

In this study, the pTT5-CTP-PTEN eukaryotic expression vector was constructed, the CTP-PTEN fusion protein was expressed and purified, and 5637 bladder cancer cells were cocultured with PTEN and CTP-PTEN fusion protein. The carcinostatic activities of PTEN and CTP-PTEN were evaluated using a variety of techniques, such as RT-PCR, WB, the MTT assay, flow cytometry, the Transwell assay, and the cell scratch assay. The results showed that both PTEN and CTP-PTEN inhibited the cell cycle, proliferation, invasion and migratory rate of 5637 cancer cells and promoted the apoptosis of 5637 cells. In addition, the expression of the Akt, mTOR and Pi3k genes were significantly downregulated. The content of PIP3 was significantly reduced in the PTEN and CTP-PTEN group. This study showed that the carcinostatic effect of CTP-PTEN might be closely related to the inhibition of the expression of mTOR pathway-related proteins. The results of this study indicated that compared with PTEN, CTP-PTEN had a more significant inhibitory effect on cancer cells. Such a finding demonstrated that the fusion expression of CTP and PTEN significantly enhanced the penetrability of the tumor suppressor gene into cancer cells.

In summary, this study provides a theoretical basis for the new bladder cancer treatments. Moreover, CTP-mediated PTEN protein might be further developed into a novel antitumor drug, which has a certain clinical value.

## Supplementary Information

**Additional file 1:** The effect of CTP-PTEN and PTEN on invasion, expression of target genes, expression and phosphorylation of proteins, content of PIP3 in 5637 cell lines.

## Data Availability

The datasets used and/or analysed during the current study available from the corresponding author on reasonable request.
