# Supplementary material for: Effect of CTP-mediated PTEN on 5637 bladder cancer cells and the underlying molecular mechanism
Source: BMC Urol. 2022 Dec 10;22:200. doi: 10.1186/s12894-022-01152-y (PMC9741776; doi:10.1186/s12894-022-01152-y)
Supplement: Supplementary file 1 — Additional file 1: The effect of CTP-PTEN and PTEN on invasion, expression of target genes, expression and phosphorylation of proteins, content of PIP3 in 5637 cell lines. [file 12894_2022_1152_MOESM1_ESM.docx]

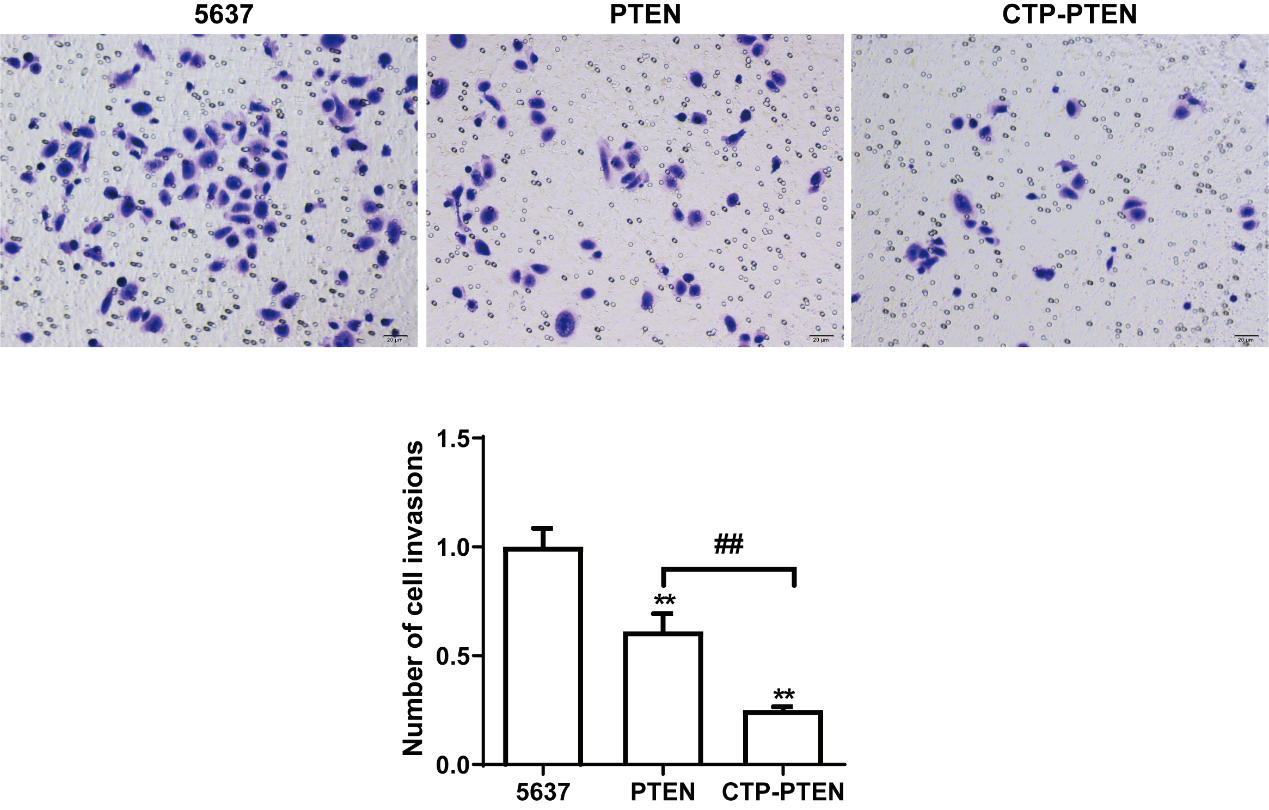


Figure S-1. The effect of CTP-PTEN and PTEN on invasion of 5637 cell lines


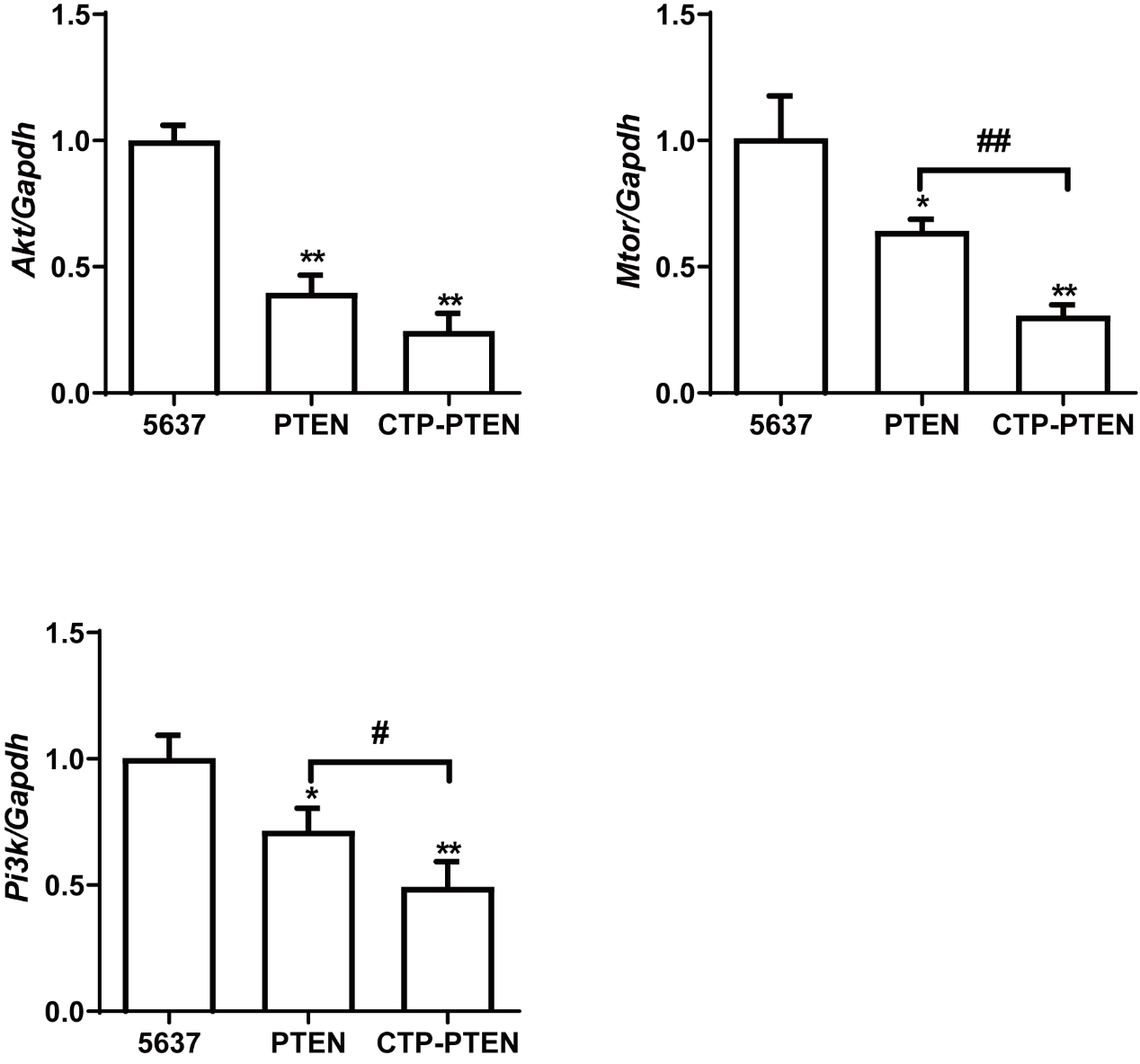


Figure S-2. The effect of CTP-PTEN and PTEN on expression of target genes in 5637 cell lines


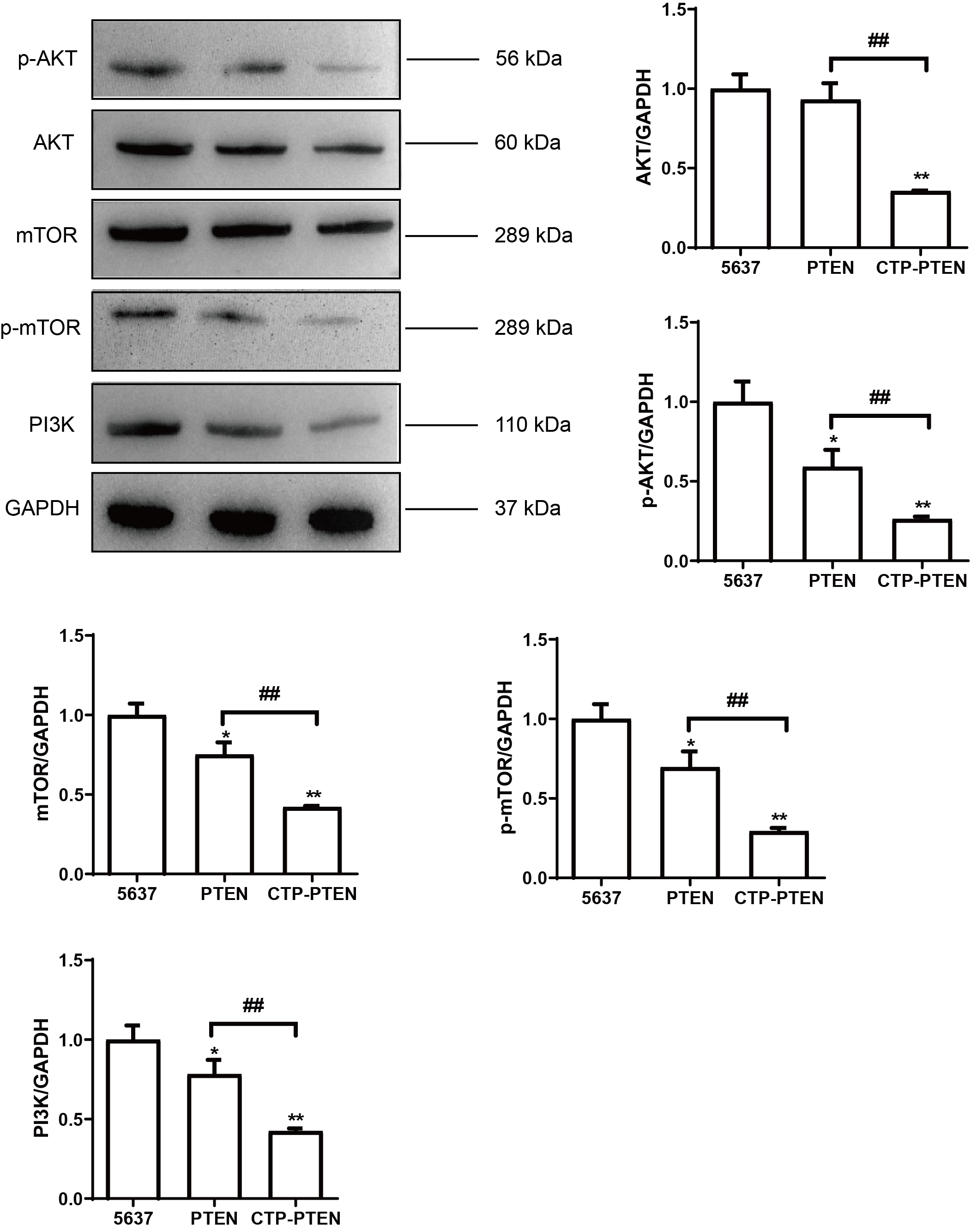


Figure S-3. The effect of CTP-PTEN and PTEN on expression and phosphorylation of proteins in 5637 cell lines


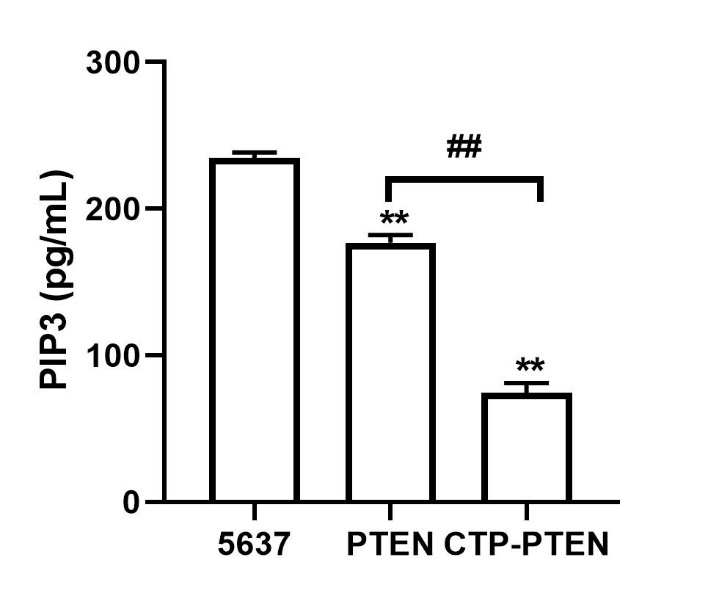


Figure S-4. The effect of CTP-PTEN and PTEN on content of PIP3 in 5637 cell lines
